# Parathyroid Hormone Secretion and Receptor Expression Determine the Age-Related Degree of Osteogenic Differentiation in Dental Pulp Stem Cells

**DOI:** 10.3390/jpm11050349

**Published:** 2021-04-27

**Authors:** Shilpa Bhandi, Ahmed Alkahtani, Rodolfo Reda, Mohammed Mashyakhy, Nezar Boreak, Prabhadevi C. Maganur, Satish Vishwanathaiah, Deepak Mehta, Nishant Vyas, Vikrant Patil, A. Thirumal Raj, Luca Testarelli, Shankargouda Patil

**Affiliations:** 1 Department of Restorative Dental Sciences, Division of Operative Dentistry, College of Dentistry, Jazan University, Jazan 45142, Saudi Arabia; shilpa.bhandi@gmail.com; 2 Department of Restorative Dental Sciences, College of Dentistry, King Saud University, Riyadh 11451, Saudi Arabia; ahkahtani@ksu.edu.sa; 3 Department of Oral and Maxillofacial Sciences, Sapienza University of Rome, 00185 Rome, Italy; rodolforeda17@gmail.com (R.R.); luca.testarelli@uniroma1.it (L.T.); 4 Department of Restorative Dental Sciences, College of Dentistry, Jazan University, Jazan 45142, Saudi Arabia; dr.mashyakhy@gmail.com (M.M.); nezarboreak@gmail.com (N.B.); 5 Department of Preventive Dental Sciences, Division of Pedodontics, Jazan University, Jazan 45142, Saudi Arabia; prabhadevi.maganur@gmail.com (P.C.M.); drvsatish77@gmail.com (S.V.); 6 Department of Preventive and Restorative Dentistry, College of Dental Medicine, University of Sharjah, Sharjah 27272, United Arab Emirates; dmehta@sharjah.ac.ae; 7 Logical Life Science Private Limited, Pune 411041, India; logicalbiology84@gmail.com (N.V.); patilvikrant.r@gmail.com (V.P.); 8 Department of Oral Pathology and Microbiology, Sri Venkateswara Dental College and Hospital, Chennai 600130, India; thirumalraj666@gmail.com; 9 Department of Maxillofacial Surgery and Diagnostic Sciences, Division of Oral Pathology, College of Dentistry, Jazan University, Jazan 45142, Saudi Arabia

**Keywords:** aging, dental pulp, osteogenesis, parathyroid hormone, stem cells

## Abstract

Objective: To demonstrate the levels of parathyroid hormone secretion and genetic expressions of parathyroid hormone (PTH) and PTH1 receptor (PTH1R) genes in the dental pulp stem cells (DPSCs) from different age groups before and after induction of osteogenic differentiation. In addition, we also wanted to check their correlation with the degree of osteogenic differentiation. Methods: Human primary DPSCs from three age groups (milk tooth (SHEDs), 7–12 years old; young DPSCs (yDPSCs), 20–40 years old; old DPSCs (oDPSCs), 60+ years old) were characterized for mesenchymal stem cell (MSC) markers. DPSCs were subjected to osteogenic differentiation and functional staining. Gene expression levels were analyzed by qRT-PCR. Surface receptor analysis was done by flow cytometry. Comparative protein levels were evaluated by ELISA. Results: All SHEDs, yDPSCs, and oDPSCs were found to be expressing mesenchymal stem cell markers. SHEDs showed more mineralization than yDPSCs and oDPSCs after osteogenic induction. SHEDs exhibited higher expression of PTH and PTH1R before and after osteogenic induction, and after osteogenic induction, SHEDs showed more expression for RUNX2, ALPL, and OCN. Higher levels of PTH were observed in SHEDs and yDPSCs, and the number of PTH1R positive cells was relatively lower in yDPSCs and oDPSCs than in SHEDs. After osteogenic induction, SHEDs were superior in the secretion of OPG, and the secretions of ALPL and PTH and the number of PTH1R positive cells were relatively low in the oDPSCs. Conclusions: The therapeutic quality of dental pulp stem cells is largely based on their ability to retain their stemness characteristics. This study emphasizes the criterion of aging, which affects the secretion of PTH by these cells, which in turn attenuates their osteogenic potential.

## 1. Introduction

Parathyroid hormone (PTH) was the first-ever drug to be approved by the Food and Drug Administration (FDA) in the U.S. for the remedy for osteoporosis. PTH promotes bone remodeling, which includes both bone resorption as well as formation. Osteoblasts have receptors for PTH known as the parathyroid hormone receptor (PTHR), to which PTH binds and stimulates phospholipase C (PLC) and protein kinase C (PKC) [1,2].

PTH has been reported to stimulate the proliferation of bone marrow mesenchymal stem cells (BMSCs) along with increased intracellular calcium fluorescence intensity and also the proliferation of human mesenchymal stem cells [3,4]. Studies have shown that PTH, when administered intermittently, shows anabolic effects whereas, when given continuously, imparts catabolic effects [2,4,5,6]. Such studies have been taken further to examine the effect of PTH on the healing properties of stem cells. There have been both in vitro and in vivo studies that point out the bone healing as well as the angiogenic properties induced by PTH on cells [7]. In one such study, it was shown that vitamin K and PTH have a synergistic effect on osteoblast differentiation and hence bone formation in rats with osteoporosis [8]. The effect of PTH is not limited to bones. Lee et al. showed that the tissue deposition can be accelerated to the tendons when administered with PTH and enhance the tendon repair process [9]. All these effects of PTH are attributed to the mechanism involving increased expression of the receptor activator of nuclear factor k-B ligand (RANKL) and macrophage colony-stimulating factor (M-CSF) [2]. Other factors such as type I collagen (COL1A1), osteocalcin, osteonectin, and bone morphogenetic protein receptor II (BMPR II) have also been found to be increased in the progenitor cells [6,7]. Another plausible mechanism for such effects lies with the migration capabilities of progenitors from the bone marrow environment to the bloodstream and hence to the site of the wound, especially induced by PTH. It might be possible that various fragments (N terminal and C terminal) of Parathyroid hormone-related peptide (PTHrP) are responsible for various activities. This is corroborated by the work of Casado-Díaz et al. [10]. They showed that the N terminus was able to induce osteogenic differentiation, whereas the C terminus induced adipogenic differentiation of mesenchymal stem cells. Such studies have made it clear that PTH has potential applications in the area of regenerative medicine, tissue engineering, transplantation, and orthodontics [11]. All these studies point towards the potential of PTH to induce osteogenic differentiation.

PTH is primarily considered to play a major role in maintaining the homeostasis of calcium. Recent studies have shown that PTH can also modulate the remodeling of bone through a combination of catabolic and anabolic effects. The type of effect induced in turn depends on the pattern of PTH administration. For example, administering PTH daily induces an anabolic effect that has been used for the treatment of osteoporosis and has U.S. Food and Drug Administration approval. Despite therapeutic approval to date, the molecular pathway of PTH-induced bone induction is not well delineated. A seven-transmembrane G-protein coupled receptor, entitled PTH type 1 receptor (PTH1R), is the target for PTH binding, which causes G-protein alpha s subunit (or) G-protein alpha q subunit activation, which in turn activates protein kinase A through cyclic adenosine monophosphate induction or activates protein kinase C through phospholipase induction. PTH augments osteogenic differentiation of MSC, although the molecular pathway remains elusive. The osteoinductive potential of PTH was largely attributed to the conglomeration of several local factors including Wnts, bone morphogenetic proteins, and transforming growth factor-beta. These factors were suggested to be gathered by recruitment of their receptors to PTH1R and/or endocytosis. For example, endocytosis can be induced on a PTH1R/TGFb type II receptor by PTH causing a tempero-spacial coupling of bone resorption and induction. Age-related attenuation of the bone mass and osteogenesis reduces the overall success of a dental implant. As mentioned earlier, at present, PTH is used in osteoporosis, and its application could potentially be extended to induce vascularized bone regeneration, which in turn could be applied for optimizing implant osseointegration in aged individuals [8,12].

Aging has been shown to modulate the osteo-microenvironment, causing trabecular bone resorption and increased bone turnover, culminating in bone loss. Studies have demonstrated an age-related attenuation of the osteogenic potential of bone marrow mesenchymal stem cells. The reduced osteogenic potential is often coupled with an augmented adipogenic potential, causing a compromised regenerative potential. Singh et al. identified that the augmented adipogenic potential could be related to the intrinsic cellular aging, which could induce adipogenic potential onto even a young transplanted bone marrow-derived mesenchymal progenitor cells. Despite the advantages of the novel implants and optimal surgical techniques, aging-induced attenuation of bone quality and quantity poses a serious risk for implant failure. Thus, modulation may be required to optimize bone regeneration, allowing adequate dental implant osseointegration [3,7].

Dental pulp stem cells (DPSCs), classified under MSCs, have a principal role of replacing damaged odontoblast with fresh ones [13]. Sakakura, in 1987, showed that on rat embryonic molars, PTH does not promote differentiation of odontoblast [14]. Additionally, there was no formation of dentin and predentin. In another similar study on mouse embryonic molars, it was again shown that PTH did not promote odontogenesis, but rather suppresses it [14]. Another study revealed that PTH does not affect the enamel formation of rat maxillary incisors [15]. Work on PTH and its effect on the embryonic tooth, molars, or maxillary incisors of rats did not consider the potential effect of PTH on mesenchymal stem cells. Recent studies have given positive results on this front. Silva et al. [16], demonstrated that intermittent administration of PTH successfully abated rat incisor eruption in rats [16]. A study concerned with PTHrP showed that, in odontoblast, for normal functioning, PTHrP is essential [17]. Similarly, it has also been concluded that PTH and PTHrP are important for the regulation of chondrocyte differentiation, which further helps in the development of other bones [18]. The maintenance and development of tooth germ via the proper function of osteoclast are directly related to PTHrP [19]. The present study aimed to demonstrate the levels of secretion and genetic expressions of PTH and PTH1R genes in the DPSCs from different age groups before and after induction of osteogenic differentiation. Additionally, the correlation with the degree of osteogenic differentiation was analyzed. The research hypothesis was that secretion and genetic expressions of PTH and PTH1R genes in the DPSCs decline with increasing age. As per the PICO framework, in the present study, the P would be the DPSC from older age groups, the I would be the tool used to assess the secretion and genetic expressions of PTH and PTH1R genes, the C would be the DPSC from the younger age groups, and the O would be the level of secretion and genetic expressions of PTH and PTH1R genes.

## 2. Materials and Methods

The scientific research (IRB) of the College of Dentistry, Jazan University, has approved the present study (reference number: CODJU19682). Culture and expansion of human DPSCs: Human dental stem cells isolated from healthy individuals exfoliated deciduous tooth (age: 8–12 years) and human premolars (age: 20–25 years and 60–70 years) were obtained from Regenerative Medicine Laboratory, Dr. D. Y. Patil Dental College and Hospital, Pune, India. The DPSCs were further expanded in DMEM (Invitrogen, Carlsbad, CA, USA) supplemented with 10% fetal bovine serum (FBS) (Gibco, Rockville, MD, USA) and antibiotic-antimycotic solution at 37 °C and 5% CO_2_. The cell growth media was replaced with fresh media every 2–3 days. At 70–80%, confluence cells were detached using 0.25% Trypsin-EDTA (Invitrogen, Carlsbad, CA, USA) solution and shifted to another flask for further expansion. Passage 4 cells were used in all experimental protocols.

Characterization using flow cytometry: The cells from the culture flasks were detached and incubated for 30 min with CD73-APC, CD90-FITC, CD105-PE, and HLA-DR-PE antibodies (Miltenyi, Bergisch Gladbach, Germany). The cells were then directly acquired on a flow cytometer (Attune NxT). About 10,000 events were acquired per sample. The amount of positively stained cells was measured as a percentage paralleling that of the isotype controls. The same method was followed for the PTH1R-APC antibody staining.

Induction of osteogenic differentiation: The cells from all the groups were subjected to osteogenic differentiation by exposing them to the osteogenic induction medium and incubated for 21 days. The medium was replaced with fresh media once every 3 days. To functionally decide the osteogenic mineralization, 4% paraformaldehyde was used to treat the cells for fixation and they were then stained for mineralization with 2% alizarin red S (pH 4.1–4.3) on day 7, day 14, and day 21, along with the uninduced controls (negative control). The quantitation of mineralization in osteoblasts stained with alizarin red was carried out by adding 4% NaOH to dissolve the stained minerals, and then the absorbance was taken at 450 nm on a spectrophotometric plate reader.

Quantitative real-time PCR to analyze the gene expression: RNA was extracted with the aid of Trizol reagent. Of the total RNA, 1 μg quantity was reverse-transcribed by treating it with cDNA synthesis reagents with the help of the guidelines by the manufacturer. Quantitative analyses of genes of interest (PTH, PTH1R, RUNX2, ALPL, and OCN) were executed by using SYBR Green PCR master mix on a qRT-PCR instrument. The expression of the target genes of interest was normalized to that of the ß-actin gene (ACTB) following the ΔΔCt approach. The data were quantified by using the 2^−ΔΔCt^ formula and indicated as normalized relative expressed genes to that of the average CT for the ACTB gene. Primers are given in Table 1.

ELISA for analysis of protein levels in the conditioned media: The analyses of protein levels of PTH, ALPL, and OPG were carried out by using commercial human ELISA kits, per the guidelines available. One hundred microliter/well samples and standards were added to the plate, and the plate was incubated at room temperature for 2 h. Then the plate was washed 4 times and 100 µL detection antibody was introduced in each well; the plate was further incubated for 2 h at room temperature. For a second time, the plate was washed 4 times. One hundred microliters of streptavidin-HRP was introduced in each well and incubated for 30 min at room temperature. After washing the plate 4 times, 100 µL of TMB substrate was added and incubated for 30 min in the dark. The reaction was stopped by adding 100 µL of stop solution. The absorbance was read spectrophotometrically at 450 nm.

Effect of L-Thyroxine on osteogenic differentiation of DPSCs: The cell density of 2500 cells/cm^2^ was used in a 24-well plate (Nunc, Rochester, NY, USA) with a complete growth medium. After 24 h, the complete growth medium was replaced with an osteogenic induction medium, which was DMEM with 1% antibiotic-antimycotic, 0.1 µM of dexamethasone, 50 µM of ascorbate-2-phosphate, and 10 mM of β-glycerophosphate (Sigma–Aldrich Corp., St. Louis, MO, USA). Four experimental groups were created; control, Osteo, and 1 μM L-Thyroxine (T4) (Sigma–Aldrich Corp., St. Louis, MO, USA) + Osteo. The medium was replaced with a fresh induction medium with the same composition twice a week. To analyze the differentiation after 21 days towards osteogenic lineage, the cells were fixed with 4% paraformaldehyde, and 2% alizarin red S (pH 4.1–4.3) staining was performed for 20 min. The quantitation of alizarin red S-stained osteoblasts was done by dissolving stained cells in 4% NaOH, and the dissolved stain was read spectrophotometrically at 405 nm.

Statistical analysis: All the data values were showed by way of mean ± standard deviation. Altogether, the respective experimental groups were compared and analyzed by using paired *t*-test (two-tailed); *p*-value < 0.05 was specified as significant (levels of significance: * *p* < 0.05 and ** *p* < 0.01).

## 3. Results

**Figure 1:**
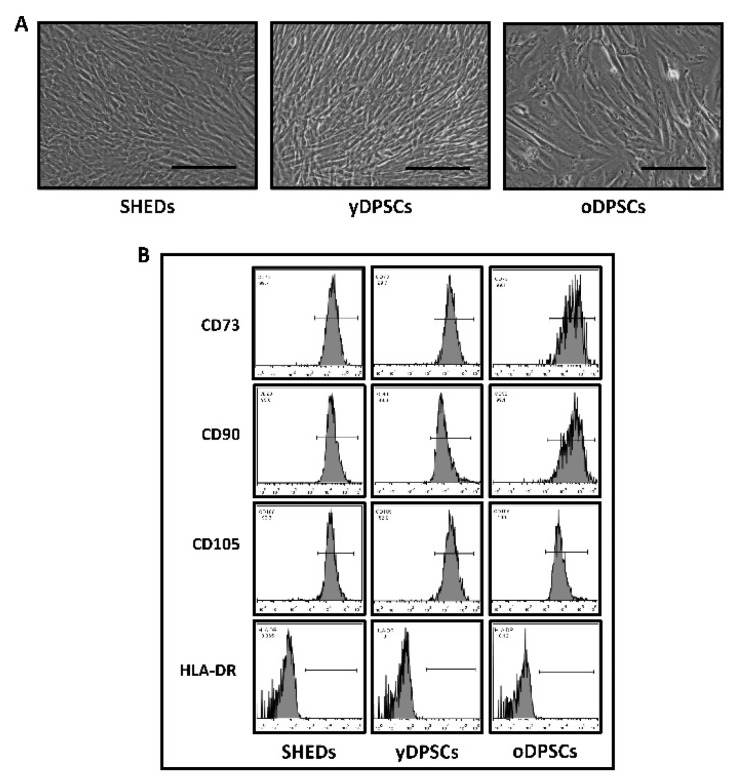
Characterization of dental pulp stem cells from different age groups. (**A**) Morphology of SHEDs, yDPSCs, and oDPSCs at Passage 4. (**B**) Analysis of a cluster of differentiation markers CD73, CD90, CD105, and HLA-DR by flow cytometry on SHEDs, yDPSCs, and oDPSCs (*n* = 5). Scale bar = 100 µm. SHEDs: Stem cells from exfoliated deciduous tooth, yDPSCs: Dental pulp stem cells from young subjects, oDPSCs: Dental pulp stem cells from old subjects.

SHEDs, yDPSCs, and oDPSCs abide by the mesenchymal stem cell-like appearance, and a cellular cluster of differentiation markers present on the cell surface. The morphological features of all the dental pulp stem cells including SHEDs, yDPSCs, and oDPSCs were examined under a microscope. They all presented an elongated spindle-shaped morphological character, which is possessed by ideal mesenchymal stem cells (Figure 1A).

**Figure 2:**
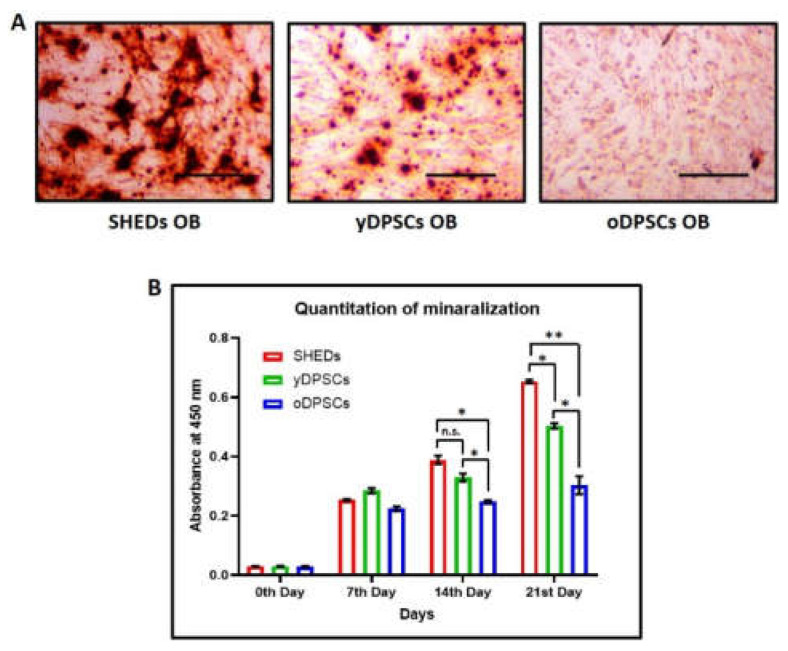
Osteogenic differentiation of SHEDs, yDPSCs, and oDPSCs. (**A**) Differentiated osteoblasts from SHEDs, yDPSCs, and oDPSCs stained for mineralization with alizarin red s. (**B**) Quantitation of alizarin red s for comparative estimation of mineralization in osteoblasts differentiated from SHEDs, yDPSCs, and oDPSCs (*n* = 5). n.s.: not significant, * *p* < 0.05, ** *p* < 0.01. SHEDs: Stem cells from exfoliated deciduous tooth, yDPSCs: Dental pulp stem cells from young subjects, oDPSCs: Dental pulp stem cells from old subjects, OB: osteoblasts.

All the dental pulp stem cells, including SHEDs, yDPSCs, and oDPSCs, showed positive expression (more than 90%) for CD73, CD90, and CD105, which are mesenchymal stem cell-specific cell-surface markers (Figure 1B); nevertheless, the expression of HLA-DR, which is an MHC class-II cell surface antigen, was found to be negative (less than 0.1%) in all dental pulp stem cells, including SHEDs, yDPSCs, and oDPSCs (Figure 1B). These results show that all the dental pulp stem cells, including SHEDs, yDPSCs, and oDPSCs, are without the contamination of any other cell type, which categorizes them as a promising source for cellular transplantation use in the field of medicine and dentistry. SHEDs show superior osteogenic differentiation and mineralization as compared to the dental pulp stem cells from young (yDPSCs) and old (oDPSCs) subjects; all the dental pulp stem cells, including SHEDs, yDPSCs, and oDPSCs, showed successful osteogenic differentiation upon induction, as assessed by the functional staining for mineralization with alizarin red (Figure 2A).

Moreover, we estimated the degree of osteogenic differentiation by the quantitation of mineralization on 3 different time points of 7 days, 14 days, and 21 days. The graphical representation of the data suggests that as the maturation of osteoblasts takes place, the SHEDs show enhanced mineralization significantly higher than that of the yDPSCs and oDPSCs (Figure 2B). It is evident from this data that aging somehow helps the stem cells to lose their osteogenic differentiation potential.

**Figure 3:**
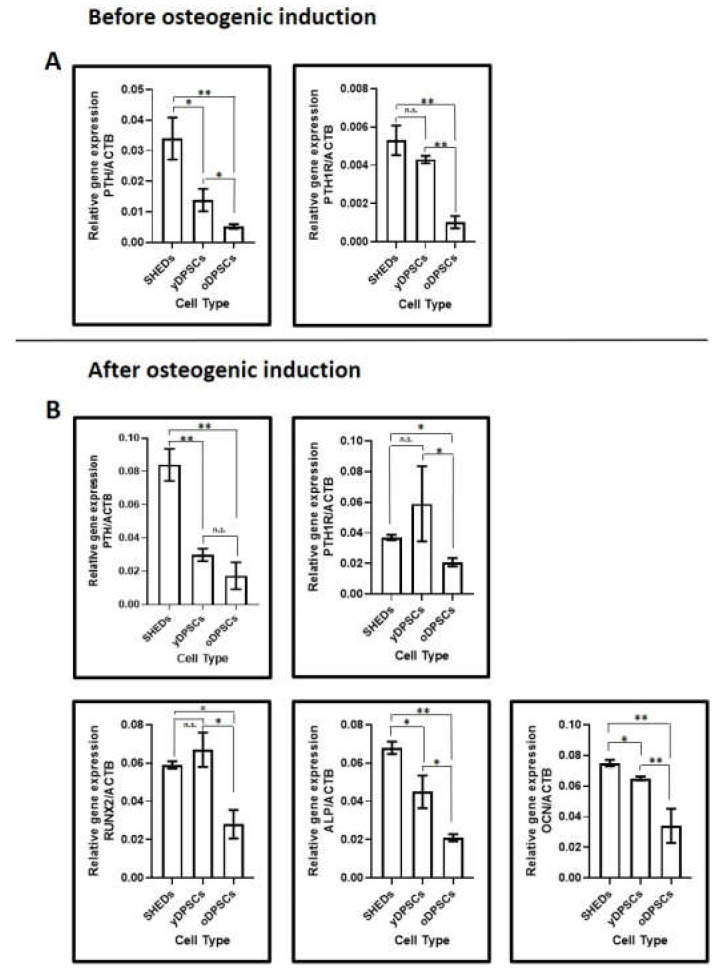
RT-qPCR analysis for estimation of gene expression. (**A**) Comparative gene expression levels of PTH and PTH1R were assessed in SHEDs, yDPSCs, and oDPSCs. (**B**) On the 14th day of osteogenic induction, comparative gene expression levels of PTH, PTH1R, RUNX2, ALP, and OCN were assessed in SHEDs, yDPSCs, and oDPSCs (*n* = 5). n.s.: not significant, * *p* < 0.05, ** *p* < 0.01. SHEDs: Stem cells from exfoliated deciduous tooth, yDPSCs: Dental pulp stem cells from young subjects, oDPSCs: Dental pulp stem cells from old subjects, OB: osteoblasts.

PTH and PTH1R are more highly expressed in SHEDs than in dental pulp stem cells from young and old subjects: Upon comparison of gene expression levels of PTH and PTH1R in all dental pulp stem cells, it was observed that SHEDs showed superior expression of PTH than yDPSCs and oDPSCs, though the levels were significantly higher in yDPSCs than oDPSCs. On the other hand, PTH-R1 expression was comparable in SHEDs and yDPSCs, and significantly lower in oDPSCs than the others (Figure 3A).

After the osteogenic induction of dental pulp stem cells, the expression of PTH and PTH1R were more elevated in the induced cells than that in those of the uninduced controls. But when we compared the cells after osteogenic induction, the same trend of PTH and PTH1R expression was observed as that of the uninduced controls (Figure 3B). Alongside, we examined the osteogenic markers after 14 days of induction, and it was undoubtedly evident that the expression of the osteogenesis-related genes RUNX2, ALPL, and OCN was significantly lower in the aged dental pulp stem cells that are oDPSC than in the SHEDs and yDPSCs induced osteoblasts (Figure 3B).

**Figure 4:**
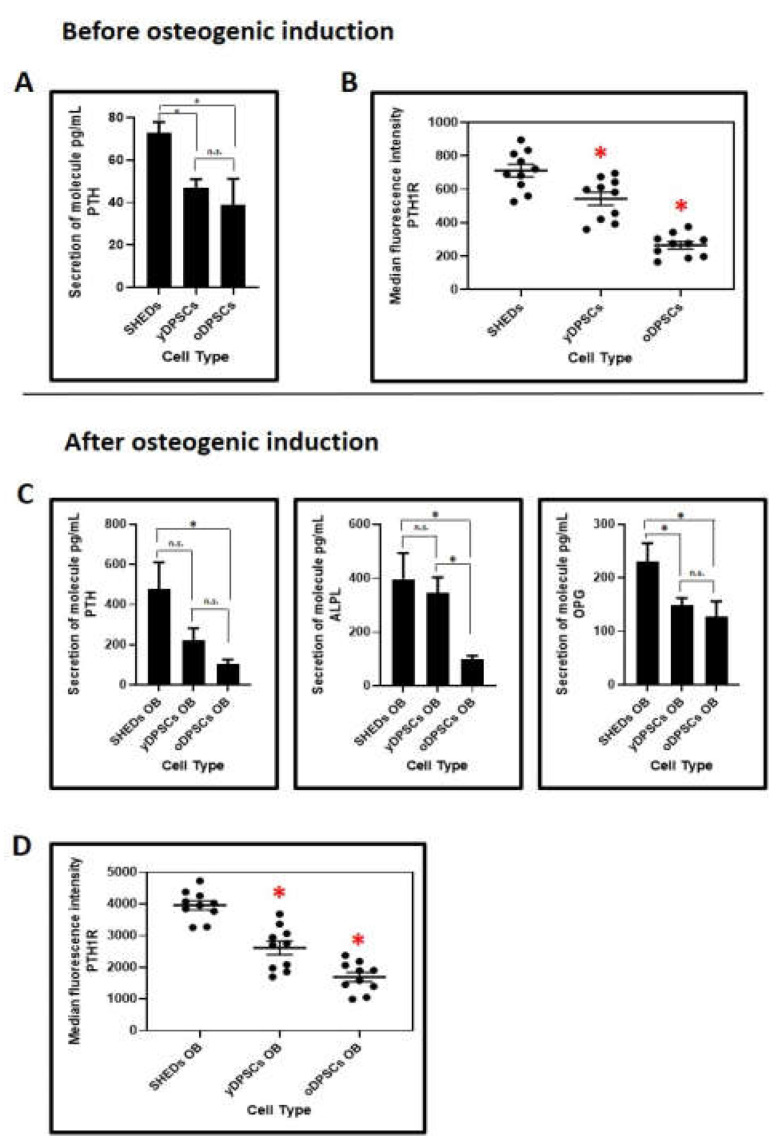
ELISA-based protein expression analyses and flow cytometry-based surface protein analysis. (**A & B**) Comparative protein expression levels of PTH and PTH1R were assessed in SHEDs, yDPSCs, and oDPSCs. (**C & D**) On the 14th day of osteogenic induction, comparative protein expression levels of PTH, PTH1R, ALPL, and OPG were assessed in SHEDs, yDPSCs, and oDPSCs (*n* = 5). n.s.: not significant, * *p* < 0.05. SHEDs: Stem cells from exfoliated deciduous tooth, yDPSCs: Dental pulp stem cells from young subjects, oDPSCs: Dental pulp stem cells from old subjects, OB: osteoblasts.

SHEDs are better in the secretion of PTH and PTH1R at the protein levels than dental pulp stem cells yDPSCs and oDPSCs: Further to the gene expression analysis, we assessed the PTH and PTH1R protein levels in the secretome of all the dental pulp stem cells including SHEDs, yDPSCs, and oDPSCs. SHEDs were secreting significantly higher levels of PTH and showed, moreover, better protein expression of PTH1R than yDPSCs and oDPSCs (Figure 4A,B).

After osteogenic induction, along with the PTH and PTH1R, we compared the protein levels of ALPL and OPG (Figure 4B,D). Though PTH and ALPL levels were comparable in the SHEDs and yDPSCs and significantly higher than in the oDPSCs, the protein OPG was secreted superbly in the SHEDs. The protein expression of PTH1R was significantly higher in the SHEDs than in the yDPSCs and oDPSCs; it was also significantly higher in yDPSCs than in oDPSCs.

**Figure 5:**
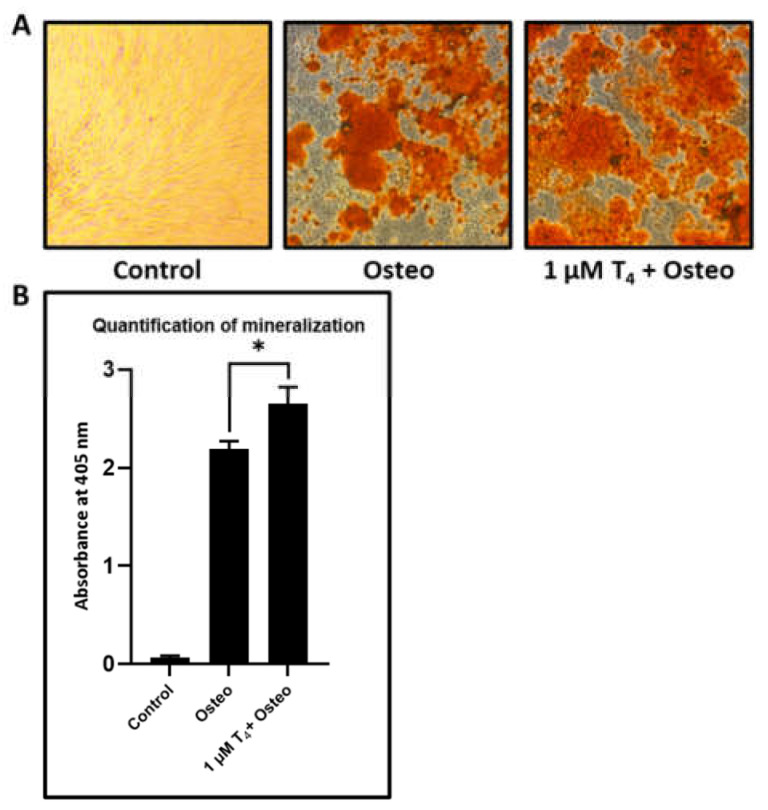
Osteogenic differentiation of DPSCs. (**A**) Differentiated osteoblasts from DPSCs and L-thyroxine treated DPSCs were stained for mineralization with alizarin red S. (**B**) Quantitation of alizarin red S for comparative estimation of mineralization in osteoblasts differentiated from DPSCs and L-thyroxine treated DPSCs (*n* = 5). * *p* < 0.05. Osteo: osteogenic induction, T4: L-thyroxine. DPSCs: Dental pulp stem cells.

L-Thyroxine shows a significant increase in the mineralization in osteogenic induced DPSCs: DPSCs were given osteogenic induction with osteogenic differentiation media and treated with L-thyroxine. After 21 days of induction, the DPSCs treated with L-thyroxine showed significantly enhanced mineralization than just osteogenically differentiated DPSCs (Figure 5A,B).

## 4. Discussion

DPSCs have a wide range of therapeutic utility in orthopedics in the field of dentistry. The principal role of DPSCs is the replacement of odontoblasts with fresh ones [13]. Currently, PTH is the only FDA-approved drug for osteoporosis in the U.S., which has a clinical use of 2 years. The present study attempted to elucidate the autocrine effects of PTH on DPSCs and their differentiation. A special emphasis is given to the criterion of aging that contributes to the osteogenic potential of these cells by affecting their PTH secretion. To that end, the PTH and PTH1R levels were assessed in the DPSCs from different age groups. Subsequently, their correlation with the degree of osteogenic differentiation was evaluated. A major challenge in clinic-ready DPSCs is the detailed assessment of the microenvironmental modulation of the DPSC-based osteogenesis and the underlying molecular mechanisms to elucidate the specific contributors to the microenvironment and the key molecules and signaling pathways involved. Thus, studying the upstream and downstream regulators of the molecules modulating the microenvironment would aid in the establishment of key ideas that determine the therapeutic utility of these stem cells. Numerous signaling molecules contribute to the complex pathways of osteogenic differentiation of these cells. Amongst these thoroughly studied molecules, PTH and PTH1R have been shown to regulate and augment the osteogenic differentiation of stem cells [7,8]. This is the first report demonstrating the regulation of osteogenic differentiation of dental pulp stem cells by PTH and PTH1R in an autocrine fashion. Moreover, we also demonstrate that aging affects the expression of PTH and PTH1R genes, in turn thwarting their protein translation.

PTH on daily administration has shown promise in augmenting bone formation and has been successful in treating osteoporosis. PTH effects were attributed to its role in regulating key osteogenic factors including BMP, TGF-β, and IGF-1, which allows osteogenic differentiation of the MSCs. Recent studies have suggested a role for PTH-like osteogenic agents in enhancing osseointegration in dental implants. A recombinant human PTH (1–34) designated as teriparatide is the sole U.S. FDA-approved anabolic agent used to augment bone mass in aging and osteoporosis. Teriparatide is shown to induce its effects through activation of the quiescent lining cells on the bone modeling surface to osteoblasts, causing induction of bone. Intermittent PTH (1–34) administration is required for augmenting bone regeneration to enhance bone-to-implant contact (BIC) in ovariectomized animal models. Prx1Cre; PTH1Rfl/fl mice model studies have shown that PTH’s therapeutic potential can be largely attributed to the modulation of the differentiation potential of the bone mesenchymal stem cells. The osteogenesis and angiogenesis noted in bone formation and remodeling are suggested to be coupled. The mechanism of how the osteoclasts react in response to PTH-induced angiogenesis or osteogenesis needs further investigation [7,8]. Silva et al. [17], in 2016, evaluated the influence of intermittent administration of PTH (1–34) on lower incisor eruption rates in rats under normal, hyper, and hypofunctional conditions. The results showed significantly reduced eruption rates in PTH-treated animals under all conditions. PTH-treated rats displayed an augmented bone formation and Sharpey fiber area density.

The different types of DPSCs, SHEDs, yDPSCs, and oDPSCs were found to show osteogenic differentiation following assessment by alizarin staining for mineralization. Mineralization is an important aspect to be considered during osteoinduction. It is considered that the formation of a mineralized callus can be correlated to the number of progenitor cells required for osteogenesis or healing of fractures [20]. Previously, poor bone mineral quality in the long-term posed a hurdle in osteoinduction with rhBMP-2 [20]. In the past, PTH has been shown to contribute to improved mineralization as well as an initial decline in mineralization [21,22]. The degree of mineralization, at 7 days, 14 days, and 21 days, was observed to be significantly higher for SHEDs when compared to yDPSCs and oDPSCs. SHEDs show a high degree of osteogenic differentiation upon induction.

Subsequently, the gene expression levels of PTH and PTH1R were evaluated using RT-PCR. PTH levels have been associated with chondrocyte and osteoblast proliferation, delayed chondrocyte hypertrophy, and rapid bone healing [23]. Even though the expression levels of PTH and PTH1R were elevated post-induction, a similar trend in the expression level was observed among the three types of cells, indicating that the genes had no significant effect on osteoinduction. The experiment showed increased levels of expression of PTH and PTH1R in SHEDs in comparison with other DPSCs.

Concurrent evaluation of osteoblast-specific genes, RUNX2, ALPL, and OCN, showed that the expression level was lower in the oDPSCs relative to the SHEDs and yDPSCs. While intermittent PTH treatment has been shown to lead to high levels of osteoblast-specific genes during osteogenic differentiation on human MSCs [3], this effect of PTH on DPSC types has never been evaluated.

Moreover, SHEDs were also observed to have higher levels of secretion of PTH and PTH1R following the assessment of protein levels in the secretome of DPSCs. The protein levels of PTH and ALPL were found to be comparable in SHEDs and yDPSCs, but lower in oDPSCs. SHEDs, however, showed an increased expression of the protein OPG. This may be due to the relatively higher osteogenic potential of SHEDs and yDPSCs. High ALPL levels have been significantly elevated during osteodifferentiation [24]. The protein level of PTH1R was observed to be significantly higher in SHEDs compared to other DPSCs.

The present study has successfully shown that aging causes a decrease in the PTH and PTH1R in dental pulp stem cells at gene expression as well as protein level in terms of secretion of PTH and expression of PTH1R on cell surface, in turn impairing the osteogenic differentiation ability of dental pulp stem cells. However, there were limitations as delineation of the underlying molecular mechanism was not elicited. The genes and proteins were assessed quantitatively, but qualitative analysis was not carried out. Moreover, a comprehensive experimental approach is necessary to delineate the intermingling signaling pathways along with other secretary molecules to exactly pinpoint the master regulators.

Further investigation could assess the role of PTH expression by dental pulp stem cells in bone metabolism and bone resorption as well. Additionally, it would be interesting to see how the difference in PTH levels due to age affects the osteoclastogenesis in these cells.

## 5. Conclusions

This study hints at the link between aging in dental pulp stem cells and the role of PTH and PTH1R in osteogenesis. These results propose that studying the upstream and downstream regulators of these molecules would possibly establish some key ideas, which would further help in establishing the deciding factor in the therapeutic use of stem cells and to improve the quality and efficacy of clinic-ready stem cells.

## Figures and Tables

**Table 1:** List of Primers.

Gene	Forward Primer	Reverse Primer
RUNX2	5′-GAC-TGT-GGT-TAC-CGT-CAT-GGC-3′	5′-ACT-TGG-TTT-TTC-ATA-ACA-GCG-GA-3′
ALPL	5′-GCT-GTA-AGG-ACA-TCG-CCT-ACC-A--3′	5′-CCT-GGC-TTT-CTC-GTC-ACT-CTC-A-3′
OCN	5′-CTC-ACA-CTC-CTC-GCC-CTA-TT-3′	5′-TTG-GAC-ACA-AAG-GCT-GCA-C-3′
PTH	5′-GGA-GAG-AGT-AGA-ATG-GCT-GCG-T-3′	5′-ATG-GCT-CTC-AAC-CAA-GAC-ATT-GTC-3′
PTH1R	5′-TCA-CCG-TAG-CTG-TGC-TCA-TCC-T--3′	5′-GAG-TAG-AGC-ACA-GCG-TCC-TTG-A-3′
ACTB	5′-AGA-GCT-ACG-AGC-TGC-CTG-AC-3′	5’-AGC-ACT-GTG-TTG-GCG-TAC-AG-3′

## Data Availability

The datasets generated during and/or analyzed during the current study are available from the corresponding author upon reasonable requests.

## Abbreviations

MSCs	Mesenchymal stem cells
SHEDs	Stem cells from exfoliated deciduous teeth
DPSCs	Dental pulp stem cells
yDPSCs	Dental pulp stem cells from young subjects
oDPSCs	Dental pulp stem cells from old subjects
Osteo	osteogenic induction
T4	L-thyroxine
OB	osteoblasts
PTH	Parathyroid hormone
PTH1R	Parathyroid hormone 1 receptor
ALPL	Alkaline phosphatase
OPG	Osteoprotegerin
